# Cold-induced [Ca^2+^]_cyt_ elevations function to support osmoregulation in marine diatoms

**DOI:** 10.1093/plphys/kiac324

**Published:** 2022-07-27

**Authors:** Friedrich H Kleiner, Katherine E Helliwell, Abdul Chrachri, Amanda Hopes, Hannah Parry-Wilson, Trupti Gaikwad, Nova Mieszkowska, Thomas Mock, Glen L Wheeler, Colin Brownlee

**Affiliations:** The Marine Biological Association of the United Kingdom, The Laboratory, Plymouth PL1 2PB, UK; School of Ocean and Earth Science, University of Southampton, Southampton SO14 3ZH, UK; The Marine Biological Association of the United Kingdom, The Laboratory, Plymouth PL1 2PB, UK; Biosciences, College of Life and Environmental Sciences, University of Exeter, Exeter EX4 4QD, UK; The Marine Biological Association of the United Kingdom, The Laboratory, Plymouth PL1 2PB, UK; School of Environmental Sciences, University of East Anglia, Norwich NR4 7TJ, UK; The Marine Biological Association of the United Kingdom, The Laboratory, Plymouth PL1 2PB, UK; School of Ocean and Earth Science, University of Southampton, Southampton SO14 3ZH, UK; The Marine Biological Association of the United Kingdom, The Laboratory, Plymouth PL1 2PB, UK; The Marine Biological Association of the United Kingdom, The Laboratory, Plymouth PL1 2PB, UK; School of Environmental Sciences, University of Liverpool, Liverpool, L69 3GP, UK; School of Environmental Sciences, University of East Anglia, Norwich NR4 7TJ, UK; The Marine Biological Association of the United Kingdom, The Laboratory, Plymouth PL1 2PB, UK; The Marine Biological Association of the United Kingdom, The Laboratory, Plymouth PL1 2PB, UK

## Abstract

Diatoms are a group of microalgae that are important primary producers in a range of open ocean, freshwater, and intertidal environments. The latter can experience substantial long- and short-term variability in temperature, from seasonal variations to rapid temperature shifts caused by tidal immersion and emersion. As temperature is a major determinant in the distribution of diatom species, their temperature sensory and response mechanisms likely have important roles in their ecological success. We examined the mechanisms diatoms use to sense rapid changes in temperature, such as those experienced in the intertidal zone. We found that the diatoms *Phaeodactylum tricornutum* and *Thalassiosira pseudonana* exhibit a transient cytosolic Ca^2+^ ([Ca^2+^]_cyt_) elevation in response to rapid cooling, similar to those observed in plant and animal cells. However, [Ca^2+^]_cyt_ elevations were not observed in response to rapid warming. The kinetics and magnitude of cold-induced [Ca^2+^]_cyt_ elevations corresponded with the rate of temperature decrease. We did not find a role for the [Ca^2+^]_cyt_ elevations in enhancing cold tolerance but showed that cold shock induces a Ca^2+^-dependent K^+^ efflux and reduces mortality of *P. tricornutum* during a simultaneous hypo-osmotic shock. As intertidal diatom species may routinely encounter simultaneous cold and hypo-osmotic shocks during tidal cycles, we propose that cold-induced Ca^2+^ signaling interacts with osmotic signaling pathways to aid in the regulation of cell volume. Our findings provide insight into the nature of temperature perception in diatoms and highlight that cross-talk between signaling pathways may play an important role in their cellular responses to multiple simultaneous stressors.

## Teaser

A calcium signaling pathway in marine diatoms is activated by cold temperature and enhances survival during simultaneous hypo-osmotic stress.

## Introduction

Diatoms are a group of silicified unicellular algae that represent one of the most important primary producers in modern oceans. They are abundant in diverse marine environments, most notably in polar and temperate upwelling regions, where they play a critical role at the base of the marine food web (Malviya et al., 2016). Diatom communities are abundant across a broad temperature range in the surface ocean from sea ice to tropical oceans. Diatoms are also important primary producers in freshwater and brackish ecosystems, where they likely encounter an even greater range of temperatures (Souffreau et al., 2010).

Global rises in surface temperature due to anthropogenic CO_2_ emissions are set to have profound influence on marine ecosystems (Gattuso et al., 2015). These future changes in our climate will also increase the variability of temperature regimes and the prevalence of extreme events, such as marine heat waves, that may co-occur with other stressors such as low pH or deoxygenation (Harley et al., 2006; Smale et al., 2019; Gruber et al., 2021). Understanding the physiological response of diatoms and other marine phytoplankton to changes in global temperature regimes is therefore of the utmost importance. Temperature has an important impact on diatom cell physiology, influencing cell size and formation of the silica frustule (Montagnes and Franklin, 2001; Svensson et al., 2014; Javaheri et al., 2015). Individual species display a thermal niche with distinct temperature growth optima that reflect their natural environment (Liang et al., 2019). The upper and lower thermal tolerance limits, rather than the optima themselves, appear to have the greatest influence on the distribution of individual diatom species (Anderson and Rynearson, 2020), with temperatures in excess of the upper thermal tolerance limits leading to a rapid increase in the rates of cell death (Baker and Geider, 2021).

Many of these studies have focused on the physiological responses of diatoms to longer term changes in temperature. However, diatoms will also experience short-term temperature variations within their natural habitat. This is particularly so for those species that inhabit intertidal rocky shores or estuarine habitats where immersion and emersion is associated with rapid and regular temperature fluctuations. Rapid temperature changes are potentially highly damaging to diatom cells, demonstrated by their much greater vulnerability to abrupt rather than gradual temperature increases (Souffreau et al., 2010). Temperature variability may also have an important influence on the ability of diatoms to adapt to their thermal niche, as *Thalassiosira pseudonana* exhibited accelerated adaptation to higher temperatures under a fluctuating temperature regime (Schaum et al., 2018). Despite the importance of thermal tolerance in diatom physiology and ecology, relatively little is known about the physiological mechanisms that allow diatoms to perceive and respond to changes in temperature, particularly during short-term fluctuations.

Many of the cellular mechanisms involved in temperature sensing in eukaryotes involve temperature-induced changes in the structure of nucleic acids, proteins, or biological membranes that lead to a range of downstream physiological responses (Sengupta and Garrity, 2013). Ca^2+^-dependent signaling mechanisms play an important role in these temperature sensing pathways. In animal cells, heat stress is associated with Ca^2+^ influx into the cytosol via the transient receptor potential (TRP) cation channel family of temperature-sensitive ion channels (Xu et al., 2002; Clapham and Miller, 2011). Ca^2+^ signaling also plays a role in sensing low temperature in animals, for example, underpinning the rapid cold hardening response of insects (Teets et al., 2013). Land plants also employ Ca^2+^ signaling mechanisms in their response to both low and high temperatures. Rapid cooling of plants induces a transient cytosolic Ca^2+^ ([Ca^2+^]_cyt_) elevation, which leads to changes in gene expression and the establishment of cold tolerance (Knight et al., 1996; Tahtiharju et al., 1997; Knight and Knight, 2012). Some plants, such as the moss *Physcomitrium*, also display [Ca^2+^]_cyt_ elevations in response to heat shock (Saidi et al., 2009). In other plants, such as Arabidopsis (*Arabidopsis thaliana*), support for the role of high temperatures in inducing [Ca^2+^]_cyt_ elevations is mixed, although Ca^2+^ elevations are observed within the chloroplast (Lenzoni and Knight, 2019). However, a recent study demonstrated that elevated temperature-induced [Ca^2+^]_cyt_ elevations in Arabidopsis leaves, but not in pollen tubes (Weigand et al., 2021). Potential temperature sensors in plants include the cold-sensitive regulator of G-protein signaling (COLD1/RGA1) complex in rice (*Oryza sativa*), which is proposed to either function as a Ca^2+^ channel or to activate other Ca^2+^ channels (Ma et al., 2015). Specific cyclic nucleotide-gated ion channels and annexins may also play a role in temperature sensing pathways, with mutant strains in *Physcomitrium*, *O. sativa*, and Arabidopsis exhibiting diminished [Ca^2+^]_cyt_ elevations in response to cold and heat shock (Cui et al., 2020; Liu et al., 2021). However, it is currently unclear whether these ion channels sense temperature directly or are activated indirectly, for example, through changes in membrane rigidity (Plieth et al., 1999) or the cytoskeleton (Pokorna et al., 2004).

Our understanding of Ca^2+^ signaling in diatoms remains in its infancy, although Ca^2+^-dependent signaling mechanisms have been identified in response to a range of environmental stimuli, such as the supply of nutrients (phosphate and iron), hypo-osmotic shock, and the detection of toxic aldehydes (Falciatore et al., 2000; Vardi et al., 2006; Helliwell et al., 2021a, 2021b). Initial experiments using *Phaeodactylum tricornutum* cells expressing the bioluminescent Ca^2+^ reporter aequorin did not detect [Ca^2+^]_cyt_ elevations in response to low (4°C) or high (37°C) temperature (Falciatore et al., 2000). More recently, genetically encoded fluorescent Ca^2+^ reporters have been successfully expressed in *P. tricornutum* and *T. pseudonana*, enabling high-resolution imaging of [Ca^2+^]_cyt_ elevations in single diatom cells (Helliwell et al., 2021a, 2021b). These advances will now allow detailed examination of diatom signaling in response to range of stimuli, including temperature.

In this study, we set out to examine the ability of diatoms to sense short-term changes in temperature. In particular, we examined whether the well-characterized [Ca^2+^]_cyt_ elevations observed in animal and plant cells in response to rapid changes in temperature were conserved in diatoms. Using the model species *P. tricornutum* and *T. pseudonana*, which can both inhabit coastal environments that experience variable temperature regimes (De Martino et al., 2007; Alverson et al., 2011), we found that diatoms consistently exhibit a [Ca^2+^]_cyt_ elevation in response to cold shock, but do not exhibit [Ca^2+^]_cyt_ elevations in response to elevated temperature. We did not find a requirement for cold shock-induced Ca^2+^ signaling in increasing tolerance to low temperatures, but found that cold shock increases tolerance to simultaneous hypo-osmotic shocks, suggesting that integration of multiple signaling inputs may contribute to an enhanced ability to respond to these environmental stimuli.

## Materials and results

### Rapid changes in temperature in intertidal environments

**Figure 1:**
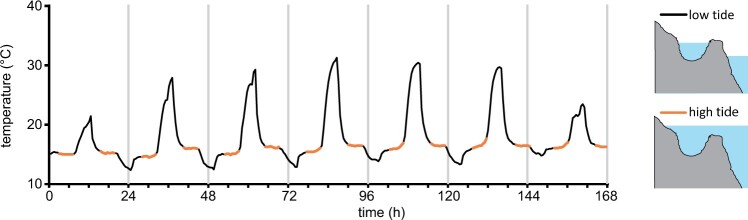
Temperature fluctuations in the intertidal zone. An example of temperature fluctuations measured in a temperate coastal rock pool (Looe, Cornwall, UK) over the course of 7 days in summer (July 1, 2019–July 07, 2019). A stable temperature was observed during periods when the pool was immersed by the high tide (approximately duration of immersion 5 h). Substantial excursions from the sea temperature occur when the rock pool is isolated from the bulk seawater at low tide (black traces). Rapid cooling (30°C–15°C) occurs when the incoming tide reaches the pool.

*Phaeodactylum tricornutum* was first isolated from a tidal pool in the UK and has since been identified in a range of coastal and brackish habitats (De Martino et al., 2007). To assess the dynamic temperature regimes potentially experienced by intertidal diatoms, we measured the temperature of a tidal pool located on the upper region of a rocky shore (South Cornwall, UK) over a 7-day period during July (UK summer). Temperatures within the pool were very stable around 15°C during immersion at high tide (Figure 1). However, at low tides temperatures in the exposed tidal pool rose substantially during the day (up to 30°C) and decreased at night (to 12°C), before being rapidly restored to the bulk seawater temperature by the immersion of the pool at high tide. These data illustrate that diatoms inhabiting intertidal environments in temperate regions will regularly experience periods of substantial warming followed by rapid cooling. The fluctuations in temperature are likely to be even greater in smaller volumes of water, such as the surface of estuarine mudflats or very shallow pools.

### Calcium signaling in response to changes in temperature

**Figure 2:**
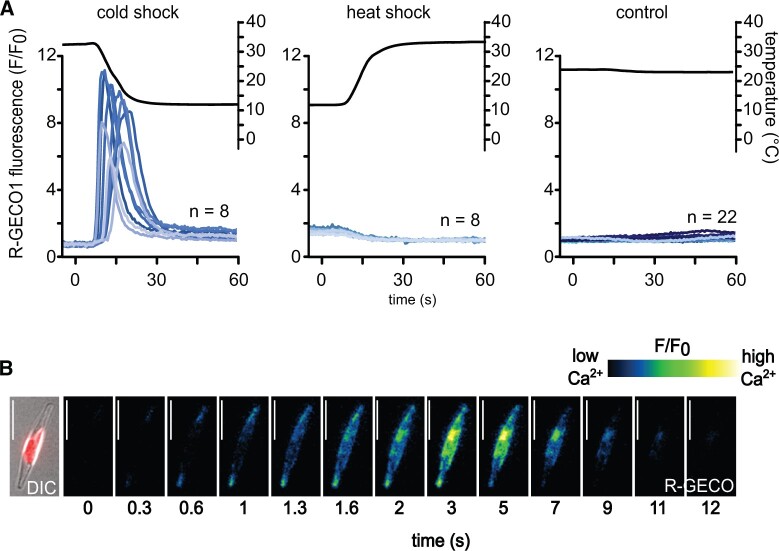
*Phaeodactylum tricornutum* exhibits cytosolic Ca^2+^ ([Ca^2+^]_cyt_) elevations in response to rapid cooling. A, Eight representative fluorescence ratio traces (*F/F*_0_, blue lines) of *P. tricornutum* cells expressing R-GECO1 representing changes in cytosolic Ca^2+^. Cells were perfused with ASW of different temperatures to cause rapid temperature shifts (black line). Cold shock 30°C–12°C, heat shock 12°C–30°C or control 22°C–22°C. B, False color images of a PtR1 cell exhibiting a [Ca^2+^]_cyt_ elevation in response to cold shock. The temperature decrease begins at *t* = 0 s. Note that the [Ca^2+^]_cyt_ elevations initiate at the tips of the cell and spread toward the central region. Left part indicates a differential interference contrast (DIC) image overlaid with chlorophyll autofluorescence. Bar represents 10 µm.

*Phaeodactylum tricornutum* cells expressing the R-GECO1 Ca^2+^ biosensor were perfused with seawater at high or low target temperatures (30°C or 12°C). Note that actual temperatures in the perfusion dish differed by ±2°C from these target temperatures due to equilibration of the small volume of warm or cold perfusate with room temperature (RT). Actual dish temperatures were therefore recorded and are displayed for all experiments. We routinely observed a single transient [Ca^2+^]_cyt_ elevation in cells exposed to a cold shock from 30°C to 12°C (97% cells, *n* = 63) (Figure 2A). In contrast, cells exposed to a rapid rise in temperature from 12°C to 30°C did not show [Ca^2+^]_cyt_ elevations (Figure 2A). No [Ca^2+^]_cyt_ elevations were observed in cells perfused with these solutions after they had been equilibrated to RT, indicating that the act of switching between the perfusion solutions does not contribute to the signaling responses (Figure 2A). Analysis of the spatial characteristics of cold shock-induced [Ca^2+^]_cyt_ elevations indicated that many initiate at the apex of the cell and propagate to the toward the central region (Figure 2B), in a manner similar to those induced by mild hypo-osmotic shock (Helliwell et al., 2021b). This suggests that the apices of the cell may play an important role in sensing the temperature changes. Cells exposed to a second cold shock 2 min after a previous cold shock demonstrated [Ca^2+^]_cyt_ elevations with no substantial attenuation in amplitude, although the percentage of cells responding was slightly lower (97%–81% of cells, *n* = 63) (Supplementary Figure S1).

The [Ca^2+^]_cyt_ elevations observed during cold shock were represented by a >10-fold increase in R-GECO1 fluorescence. Assuming a *K*_d_ of 480 nM for R-GECO1 and comparison with published maximum *F/F*_0_ values (Zhao et al., 2011), we estimate that [Ca^2+^]_cyt_ elevations reach concentrations in the micromolar range. In addition to these large increases in fluorescence that are attributed to [Ca^2+^]_cyt_ elevations, much smaller changes in the baseline fluorescence of each cell could be observed following changes in temperature (increasing with low temperature and decreasing with high temperature, Supplemental Figure S1). These minor changes most likely represent temperature-dependent changes in R-GECO1 fluorescence emission (Ohkura et al., 2012) rather than actual changes in resting Ca^2+^ concentration. Therefore, only the substantial transient increases in fluorescence (*F/F_0_* >1.5) representing large [Ca^2+^]_cyt_ elevations were analyzed further.

[Ca^2+^]_cyt_ elevations were also observed when a cold shock (30°C–12°C) was applied to cells held at 22°C, rather than 30°C, indicating that the cold shock response was not a consequence of prior warming of the cells (Supplemental Figure S2). The percentage of cells responding to cold shock was lower in cells held at 22°C compared to cells held at 30°C, although this may also be influenced by the lower maximum rate of cooling at 22°C.

### Rapid cooling is required to elicit a [Ca^2+^]_cyt_ elevation

**Figure 3:**
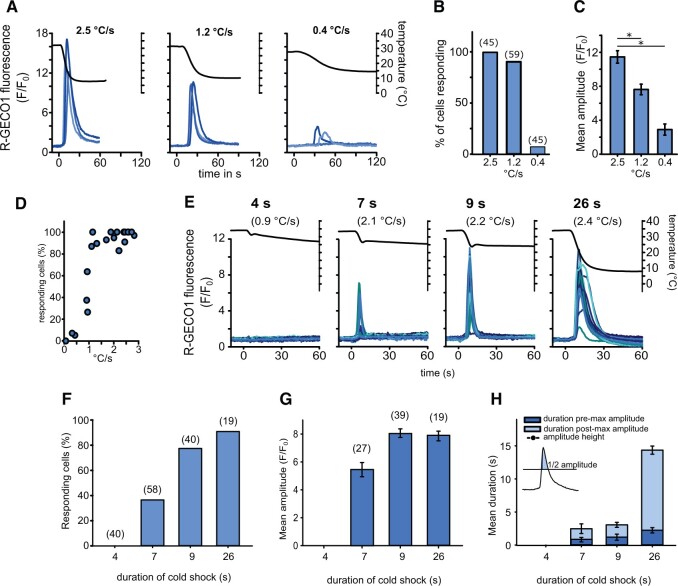
The cold shock Ca^2+^ response depends on the rate of change of temperature. A, R-GECO1 fluorescence in *P. tricornutum* in response to cold shock administered at different cooling rates. As cooling rates were nonlinear the maximal cooling rate for each treatment was calculated for comparisons. Three representative traces are shown. B, The percentage of cells exhibiting a [Ca^2+^]_cyt_ elevation (*F/F*_0_ > 1.5) at different cooling rates. Total number of cells examined are shown in parentheses, from a minimum of two separate experimental treatments. C, Mean maximal amplitude of [Ca^2+^]_cyt_ elevations from responsive cells in (B). Asterisk indicates a significant difference (one-way ANOVA on Ranks *P* > 0.001, Dunn’s post hoc test *P* > 0.001). *n* = 31, 30, and 3 for 2.5, 1.2, and 0.4°C s^−1^, respectively. Error bars = se. D, Percentage of cells responding to cold shock with a [Ca^2+^]_cyt_ elevation across a broader range of maximum cooling rates. The data represent 21 independent experiments, with a mean of 38 cells examined for each data point (minimum 12, maximum 123 cells). E, [Ca^2+^]_cyt_ elevations in response to different durations of cooling applied with a constant flow rate (16 mL min^−1^). Twenty representative traces from PtR1 cells are shown, with greater [Ca^2+^]_cyt_ elevations observed under increasing durations of cold shock. The maximum rate of temperature decrease (ΔT s^−1^) is shown in parentheses. Data for 4, 7, 9, and 26 s of cold shock duration were compiled from 2, 3, 2, and 1 individual experiments, respectively. F, The percentage of cells exhibiting a [Ca^2+^]_cyt_ elevation in response to cold shock for the experiment described in (E). G, Mean maximal amplitude of [Ca^2+^]_cyt_ elevations in response to cold shock for the responding cells shown in (F). Error bars = se. H, Duration of [Ca^2+^]_cyt_ elevations (shown as full width at half maximum amplitude) in relation of the duration of cold stimulus. The duration of [Ca^2+^]_cyt_ elevations is greatest at the 26-s cold shock. The duration is divided into a pre- and post-maximal amplitude component to show that the post-maximal amplitude (tail) components of the [Ca^2+^]_cyt_ elevation is greatly extended under the 26-s cold shock. Error bars = se.

We therefore examined the nature of the temperature change required to elicit [Ca^2+^]_cyt_ elevations, by manipulating the flow rate of the perfusion to vary the rate of cooling. Rapid cooling (2.5°C s^−1^) resulted in [Ca^2+^]_cyt_ elevations in 100% of cells examined (*n* = 45), whereas only 7% of cells exhibited a [Ca^2+^]_cyt_ elevation at a cooling rate of 0.4°C s^−1^ (*n* = 45) (Figure 3A and B). The amplitude of the [Ca^2+^]_cyt_ elevations in responding cells closely corresponded with the cooling rate, with much larger [Ca^2+^]_cyt_ elevations observed at rapid cooling rates (Figure 3C). Examination of a broader range of cooling rates indicated that a cooling rate >1°C s^−1^ was required to elicit [Ca^2+^]_cyt_ elevations in 50% of the population (Figure 3D). These data suggest that the cold shock-induced [Ca^2+^]_cyt_ elevations can therefore relay information relating to the nature of the stimulus both in terms of the number of cells responding and the nature of the [Ca^2+^]_cyt_ elevation itself.

As very low perfusion rates also resulted in a lower overall decrease in temperature (due to equilibration of the perfusate with RT), we next examined the absolute temperature decrease required to initiate signaling. Cells were perfused at 30°C for 1 min and then perfused at a constant flow rate with cold artificial seawater (ASW) (4°C) for different durations to vary the decrease in temperature whilst maintaining similar rates of cooling. A very brief perfusion (4 s) lowered the temperature by 2.4 ± 0.6°C but did not induce [Ca^2+^]_cyt_ elevations (Figure 3, E and F). However, the rate of cooling in this treatment was considerably lower than the other treatments, due to buffering of the temperature by the residual volume within the perfusion dish (1 mL). Perfusions of a longer duration (7–26 s) resulted in a consistent cooling rate of 2.1–2.4°C s^−1^. A temperature decrease of 8.8 ± 0.4°C induced a [Ca^2+^]_cyt_ elevation in 38.4% of cells (*n* = 126), whereas greater decreases in temperature resulted in [Ca^2+^]_cyt_ elevations in nearly all cells (Figure 3, E and F). The amplitude and duration of [Ca^2+^]_cyt_ elevations increased with the greater duration of the temperature decrease (Figure 3, G and H). A cooling duration of 26 s did not increase the amplitude of the [Ca^2+^]_cyt_ elevation beyond those observed at 9 s, but greatly increased the duration of the [Ca^2+^]_cyt_ elevation (Figure 3, G and H). Taken together, our results show that the cooling rate and the duration of the cold shock influence the amplitude and duration of the [Ca^2+^]_cyt_ elevation and the percentage of cells responding.

### The cold shock response is conserved in the centric diatom *T. pseudonana* but displays different characteristics

**Figure 4:**
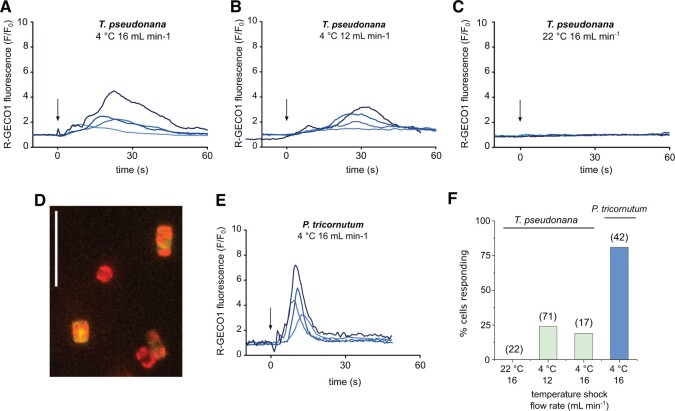
*Thalassiosira pseudonana* also shows cold-induced [Ca^2+^]_cyt_ elevations. A, Fluorescence ratio of *T. pseudonana* cells expressing cytosolic R-GECO1 in response to a cold shock (from 30°C to 10°C). For these experiments the temperature in the dish was not monitored, so perfusion flow rate is shown to indicate rate of cold shock. Arrow indicates onset of cold stimulus. Four representative traces are shown. B, As in (A) but at a slower flow rate. C, Treatment control using perfusion of ASW at RT. D, Fluorescence image of *T. pseudonana* cells expressing R-GECO1 overlaid with chlorophyll autofluorescence. Scale bar represents 20 µm. E, *Phaeodactylum tricornutum* cold shock response under identical treatment as in A for comparison. F, Percentage of cells exhibiting [Ca^2+^]_cyt_ elevations. Values in parentheses denote *n*.

*Thalassiosira pseudonana* is a planktonic centric diatom found in marine, estuarine, and freshwater environments (Alverson et al., 2011), where it is also likely to be exposed to substantial changes in temperature. We found that *T. pseudonana* cells expressing the R-GECO1 biosensor exhibited [Ca^2+^]_cyt_ elevations in response to cold shock, with the amplitude of these elevations also dependent on the rate of temperature decrease (Figure 4, A and B). As in *P. tricornutum*, a control perfusion using ASW media equilibrated to RT did not induce [Ca^2+^]_cyt_ elevations (Figure 4C). The cold-induced [Ca^2+^]_cyt_ elevations in *T. pseudonana* were of a longer duration than those observed in *P. tricornutum*, with a slower rise and fall in [Ca^2+^]_cyt_. The percentage of *T. pseudonana* cells responding to cold shock was also considerably lower than *P. tricornutum* (19% versus 81%, respectively), although variable levels of expression of R-GECO1 in *T. pseudonana* likely prevented detection of [Ca^2+^]_cyt_ elevations in all cells within a field of view (Helliwell et al., 2021a) (Figure 4D–F). Taken together, these findings suggest that cold shock-induced [Ca^2+^]_cyt_ elevations are exhibited by both pennate and centric diatom lineages and may therefore represent a conserved mechanism in many diatom species.

### Cellular mechanisms underlying the cold shock response

**Figure 5:**
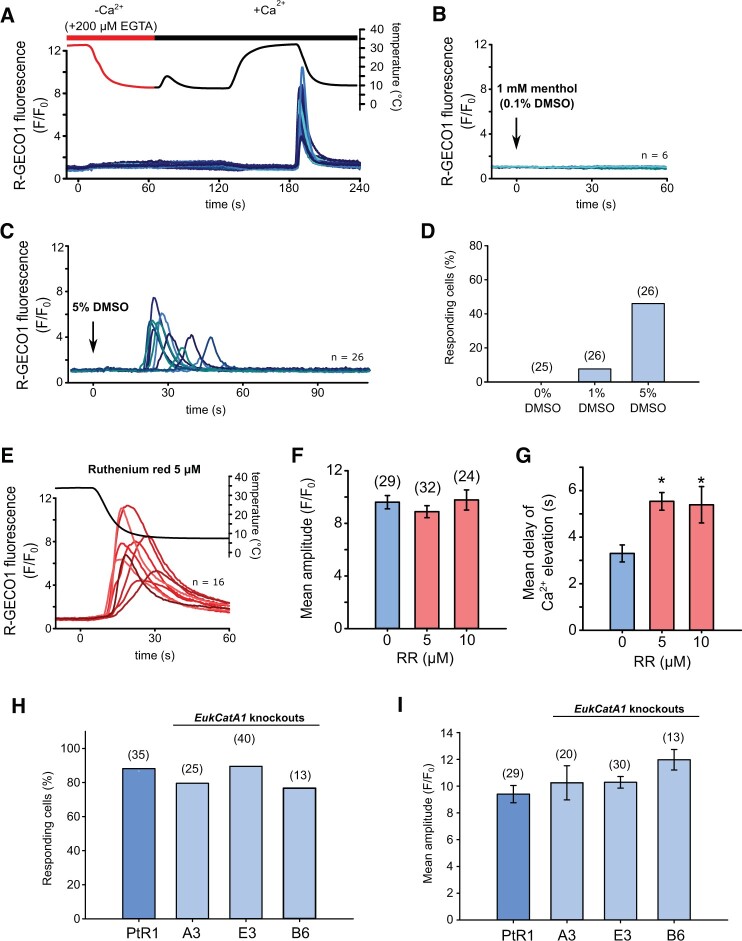
Cellular mechanisms of cold shock-induced [Ca^2+^]_cyt_ elevations. A, R-GECO1 fluorescence ratio (*F/F*_0_) from a cold shock applied to PtR1 cells using ASW without Ca^2+^ + 200-μM EGTA (Methods). No [Ca^2+^]_cyt_ elevations can be observed during the cold shock or when Ca^2+^ was restored to cold cells (perfused with cold ASW with Ca^2+^). However, [Ca^2+^]_cyt_ elevations were observed during a subsequent cold shock applied with standard ASW (i.e. with 10-mM CaCl_2_). Note the minor temperature increase at 70 s is due to a slight warming of cold ASW+Ca^2+^ media in the perfusion system. Twenty-three representative traces are shown, three additional experiments were performed with identical results. B, PtR1 fluorescence in response to ASW containing 1-mM menthol (including 0.1% DMSO as solvent carrier). Six representative traces are shown. C, R-GECO1 fluorescence ratio of PtR1 cells perfused with ASW + 5% DMSO. D, Percentage of cells exhibiting [Ca^2+^]_cyt_ elevations in response to DMSO. E, The effect of cold shock on PtR1 cells pre-treated with the Ca^2+^ channel blocker RR (5-μM final, 5-min pre-incubation). Sixteen representative traces are shown. F, Mean amplitude (±se) of responding cells treated with RR (5 μM or 10 μM) compared to untreated control cells. No significant differences were observed (*P* > 0.05, one-way ANOVA). Number of replicates is shown in parentheses. G, Mean timing (±se) of the maximal amplitude of [Ca^2+^]_cyt_ elevations for cells shown in (F) treated with RR (*P* < 0.01, one-way ANOVA, Holm–Sidak post hoc test). H, Percentage of cells showing [Ca^2+^]_cyt_ elevations in response to cold shock in control and three independent *eukcata1* mutant strains (30°C–10°C). Data represent a minimum of two independent experiments per strain. No significant differences were observed (*P* > 0.05, one-way ANOVA). I, Mean maximal amplitude (±se) of [Ca^2+^]_cyt_ elevations in *eukcatA1* mutants in response to cold shock. No significant differences were observed (*P* > 0.05, one-way ANOVA on Ranks).

We next examined the cellular mechanisms responsible for cold shock Ca^2+^ signaling in *P. tricornutum*. Removal of external Ca^2+^ by perfusion of PtR1 cells with cold Ca^2+^-free ASW completely abolished the [Ca^2+^]_cyt_ elevations (Figure 5A). Restoration of external Ca^2+^ to cooled cells did not induce a [Ca^2+^]_cyt_ elevation. However, when these cells were subsequently warmed to 30°C and then cooled, [Ca^2+^]_cyt_ elevations were observed in the majority of cells. Thus, the generation of cold-induced [Ca^2+^]_cyt_ elevation depends on the presence of external Ca^2+^, and the [Ca^2+^]_cyt_ elevation is triggered by the rapid drop in temperature rather than low absolute temperature itself.

*Phaeodactylum tricornutum* lacks cyclic-gated nucleotide channels, which are important for thermal sensing in plants, although it does possess multiple TRP channels (Verret et al., 2010). The temperature-sensitive TRP cation channel subfamily M 8 (TRPM8) in animal cells is responsible for cold-induced [Ca^2+^]_cyt_ elevations and can be activated directly by the plant secondary metabolite, menthol, at micromolar concentrations (Peier et al., 2002; Yin et al., 2018). Perfusion of PtR1 cells with 1-mM menthol did not elicit [Ca^2+^]_cyt_ elevations, indicating that this ligand is likely specific to the ion channels involved in animal cold signaling (Figure 5B). In plant and fungal cells, cold shock-induced [Ca^2+^]_cyt_ elevations have been studied through the application of dimethyl sulfoxide (DMSO), which is proposed to mimic cold-induced membrane rigidification (Furuya et al., 2014). DMSO elicited [Ca^2+^]_cyt_ elevations in a dose-dependent manner in *P. tricornutum*, with 8% and 50% of cells exhibiting Ca^2+^ elevation in response to addition of 1% and 5% DMSO, respectively (*n* = 24, 25) (Figure 5, C and D). Ruthenium red (RR) is a nonselective Ca^2+^ channel blocker shown to affect numerous TRP channels including the cold-sensitive TRPA1 channel (Andrade et al., 2008; Silva et al., 2015; Christensen et al., 2016). RR also inhibits [Ca^2+^]_cyt_ elevations in *P. tricornutum* induced by the resupply of phosphate to phosphate-limited cells, but does not inhibit [Ca^2+^]_cyt_ elevations caused by hypo-osmotic shock (Helliwell et al., 2021a, 2021b). Pretreatment of PtR1 cells for 5 min with 5–10 μM RR did not significantly reduce the amplitude of cold-induced Ca^2+^ elevations (Figure 5, E and F). However, RR-treated cells exhibited a significantly slower response time than nontreated control cells (defined as time from stimulus to the initial elevation above the threshold of *F/F*_0_ >1.5) (Figure 5G), indicating that while RR does not prevent the cold shock response, it may partially inhibit a component of the signaling pathway.

*Phaeodactylum tricornutum* contains three EukCatA channels, which represent a novel class of voltage-gated Na^+^/Ca^2+^ channel in eukaryotes related to single-domain voltage-gated Na^+^ channels in bacteria (BacNav) (Helliwell et al., 2019). As BacNav channels are temperature sensitive (Arrigoni et al., 2016), we examined whether *P. tricornutum* strains with knockout mutations in *EUKCATA1* (deletions of 38–124 bp in the region of the gene encoding the pore of the ion channel) exhibited an altered response to cold shock. The percentage of responding cells and the mean maximal amplitude of the [Ca^2+^]_cyt_ elevations did not differ significantly from control PtR1 cells (Figure 5, H and I), indicating that EukCatA1 is not required for cold shock-induced Ca^2+^ signaling. Since EukCatA1 mutants are unable to generate [Ca^2+^]_cyt_ elevations in response to membrane potential depolarization (Helliwell et al., 2019), this result also indicates that [Ca^2+^]_cyt_ elevations in response to cold or osmotic shock are independent of membrane depolarization. Further experiments will be needed to determine whether other candidate ion channels, such as the other EukCatA channels or the diatom TRP channels, contribute to the cold signaling response.

### The cold shock response is not required for growth at low temperatures

**Figure 6:**
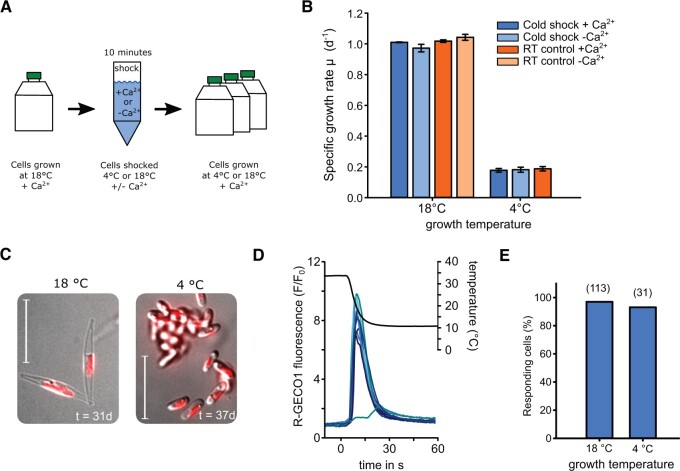
The role of the cold shock response in cold tolerance. A, Schematic diagram showing the workflow for an experiment examining the impact of Ca^2+^-dependent cold shock signaling on *P. tricornutum* cold tolerance. Cells were harvested and washed in ASW containing 10-mM Ca^2+^ or no Ca^2+^ (ASW-Ca^2+^ +200-µM EGTA). Cells were pelleted once again and exposed to cold shock with or without Ca^2+^. Cells were then grown at control (18°C) and cold (4°C) conditions in standard ASW (i.e. with 10-mM CaCl_2_) to examine cold tolerance. B, Growth rate of *P. tricornutum* cultures after cold shock treatment. Mean specific growth rates were calculated from Days 0–5 and 12–30 for 18°C and 4°C, respectively. Note that for growth at 4°C, a RT shock control in the absence of Ca^2+^ was not included. No significant differences were observed between treatments at each temperature (*P* > 0.05, one-way ANOVA). *n* = 3. Error bars represent se. C, DIC images of PtR1 cells grown at 18 or 4°C. Oval cells predominate in cells grown at 4°C for extended periods. Chlorophyll auto-fluorescence is also shown, bar = 20 µm. D, Cold-acclimated PtR1 cells still exhibit a response to cold shock. Representative R-GECO1 fluorescence ratio traces from PtR1 cells grown at 4°C for 4 days. Cells were briefly warmed to 30°C before a cold shock was applied. Traces from 15 representative cells are shown. E, The percentage of cells responding to cold shock as function of acclimation temperature. The results were generated from four separate experiments with maximum temperature drop-rates between 2.2 and 3.2C° s^−1^. Number of replicates is shown in parentheses.

We next examined whether the cold shock Ca^2+^ signal was required for *P. tricornutum* cells to survive following a cold shock. We applied cold shocks to cells in the absence of external Ca^2+^ to inhibit the cold-induced [Ca^2+^]_cyt_ elevations during cooling and then returned cells into ASW + Ca^2+^ to monitor their growth at either 4°C or 18°C (Figure 6A). While cells grew much more slowly at 4°C versus 18°C, there was no substantial impact of inhibiting Ca^2+^ signaling during cold shock on the ability of cold-shocked cells to grow at either 4°C or 18°C (Figure 6B).

Growth of *P. tricornutum* at 4°C promoted the accumulation of the oval morphotype, as reported previously (De Martino et al., 2011) (Figure 6C). Cells acclimated to low temperatures may, therefore, undergo physiological changes that render them less sensitive to rapid cooling. However, fusiform cells grown at 4°C for 4 days still showed a typical cold shock response with no substantial difference in the percentage of cells exhibiting a response (Figure 6, D and E).

Taken together, these experiments do not indicate a direct requirement for the [Ca^2+^]_cyt_ elevations in protection from rapid cooling alone, as inhibition of the signaling response did not adversely affect growth following a cold shock, and the signaling response was not altered in cells acclimated to low temperatures.

### Interaction between cold and hypo-osmotic shock Ca^2+^ signaling pathways

Diatoms inhabiting intertidal regions may regularly experience a cold shock during tidal cycles (Figure 1), but this is unlikely to represent an isolated stressor. In particular, warming of shallow tidal pools can greatly increase their salinity due to evaporation (Firth and Williams, 2009), leading to a substantial hypo-osmotic shock when the incoming tide reaches the tidal pool. *Phaeodactylum tricornutum* is highly perceptive to hypo-osmotic shock, exhibiting a large transient [Ca^2+^]_cyt_ elevation similar to those induced by cold shock (Falciatore et al., 2000; Helliwell et al., 2021b). Since cold and hypo-osmotic shocks are likely to regularly co-occur in intertidal environments, we examined cellular Ca^2+^ signaling when these stressors were applied simultaneously.

**Figure 7:**
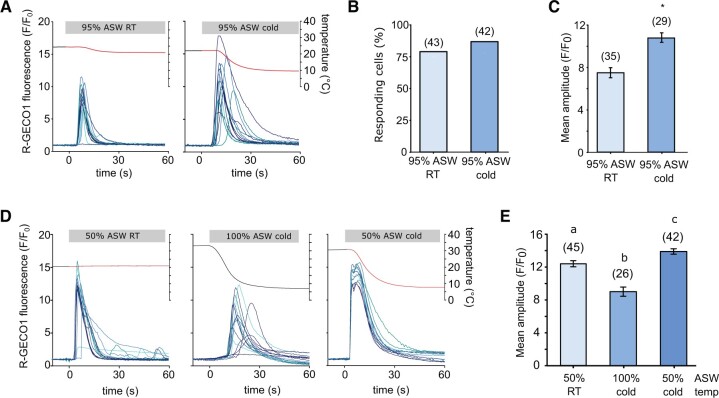
Interactions between the cold shock and hypo-osmotic shock Ca^2+^ signaling pathways. (A) R-GECO1 fluorescence ratio (*F/F*_0_) of PtR1 cells in response to a mild hypo-osmotic shock (95% ASW, left) or a simultaneous hypo-osmotic and cold shock (10°C decrease, right). Twelve representative traces are shown. B, Percentage of cells exhibiting [Ca^2+^]_cyt_ elevations for the experiment described in (A). Data are compiled from a minimum of two independent treatments. Number of replicates is shown in parentheses. C, Mean amplitude (±se) of [Ca^2+^]_cyt_ elevations from responding cells in (B). The two treatments are significantly different (Student’s *t* test *P* < 0.001). Number of replicates is shown in parentheses. D, R-GECO1 fluorescence ratio of PtR1 cells in response to stronger simultaneous cold- and hypo-osmotic shocks. Cells were treated with a single hypo-osmotic shock (50% ASW), a single cold shock (10°C) or a simultaneous cold- and hypo-osmotic shock (50% ASW, 10°C). Thirteen representative traces are shown. E, Mean maximal amplitude (±se) of cells exhibiting [Ca^2+^]_cyt_ elevations in (D). For biphasic peaks the higher amplitude was chosen. The data represent the combination of at least three independent experiments per treatment. Letters represent significant differences between treatments (one-way Kruskal–Wallis ANOVA Ranks *P* < 0.001, with Dunn post hoc). Number of replicates is shown in parentheses.

A relatively mild hypo-osmotic shock (100% ASW to 95% ASW) administered to cells at 25°C resulted in a single [Ca^2+^]_cyt_ elevation, as observed previously (Helliwell et al., 2021b) (Figure 7A). When the same hypo-osmotic shock was applied simultaneously with a cold shock (25°C–10°C), both the amplitude and duration of the [Ca^2+^]_cyt_ elevations was substantially increased, although the number of cells exhibiting [Ca^2+^]_cyt_ elevations did not change (Figure 7, A–C). Hypo-osmotic shocks cause an increase in cell volume in *P. tricornutum*, which likely initiates [Ca^2+^]_cyt_ elevations through the activation of mechanosensitive ion channels (Helliwell et al., 2021b). However, cell volume did not increase during cold shock (Supplemental Figure S3), indicating that the rapid cooling does not simply elicit [Ca^2+^]_cyt_ elevations by mimicking a hypo-osmotic stimulus.

A stronger hypo-osmotic shock (100% ASW to 50% ASW) resulted in a rapid [Ca^2+^]_cyt_ elevation which initiated directly after the stimulus was applied (Figure 7D). In comparison, application of a cold shock from 34°C to 8°C triggered [Ca^2+^]_cyt_ elevations that rose less rapidly and exhibited a longer delay to their initiation (Figure 7D). Combining both shocks using perfusion with 50% ASW at 10°C led to biphasic [Ca^2+^]_cyt_ elevations in 71% of cells (42 cells, three separate experiments) (Figure 7D). These consisted of a very rapid initial peak in [Ca^2+^]_cyt_, followed by a second peak around 3 s later, which was of greater amplitude than the first peak in the majority of cells (24 out of 30). The mean maximal amplitude of the [Ca^2+^]_cyt_ elevations caused by the three different treatments were all significantly different from each other, with the cold shock alone causing the lowest and the combined cold- and hypo-osmotic shock causing the highest [Ca^2+^]_cyt_ elevations (Figure 7E).

Taken together, [Ca^2+^]_cyt_ elevations induced by hypo-osmotic shock exhibit significant differences in amplitude and timing in the presence of a simultaneous cold shock. This indicates that the cold shock stimulus is additive and of sufficient magnitude to influence cellular Ca^2+^ signaling during hypo-osmotic stress. We therefore investigated whether Ca^2+^ signaling during cold shock may influence the short-term survival of *P. tricornutum* under hypo-osmotic stress.

### Simultaneous cold shock enhances survival during hypo-osmotic shock

**Figure 8:**
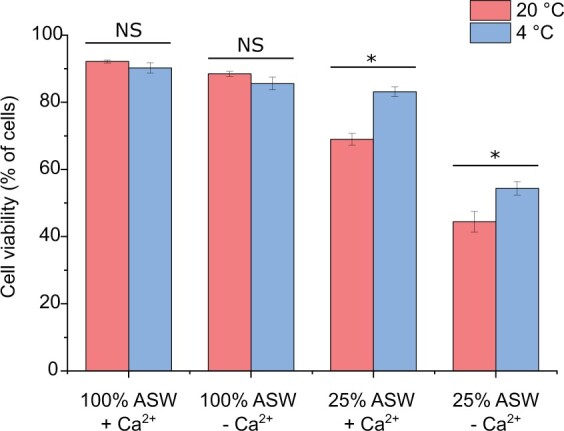
Simultaneous cold shock reduces mortality associated with hypo-osmotic shock. Cell viability (measured by exclusion of Sytox Green stain) was determined in *P. tricornutum* cells 3 min after exposure to a severe hypo-osmotic shock (25% ASW) with or without simultaneous cold shock (4°C ASW). The presence or absence of external Ca^2+^ was used to establish the effect of inhibiting Ca^2+^ signaling during the applied shocks. The decrease in cell viability due to a hypo-osmotic shock (25% ASW) is significantly reduced when a simultaneous cold shock is applied. Three replicates were performed for each treatment, with at least 100 cells counted for each replicate. Significant differences due to temperature are marked with (*P < 0.05 one-way ANOVA with Holm–Sidak post hoc test). The experiment was repeated four times with similar results each time. Error bars represent se.

Cells were treated with 25% ASW to administer a strong hypo-osmotic shock at control and low temperatures in the presence or absence of external Ca^2+^. Cell viability was determined after 3 min by staining with Sytox-Green. Administration of a cold shock alone, either in the presence or absence of external Ca^2+^, did not reduce cell viability (Figure 8). Application of a strong hypo-osmotic shock (25% ASW) significantly reduced cell viability, and this effect was greater following the removal of external Ca^2+^, supporting our previous observations that Ca^2+^ signaling is required for osmoregulation and volume control in *P. tricornutum* (Helliwell et al., 2021b). Surprisingly, application of 25% ASW in combination with a cold shock (4°C) led to a substantial reduction in cell mortality caused by hypo-osmotic shock (compared to the control temperature, 21°C). This effect was reduced by inhibiting Ca^2+^ signaling, although cell viability remained higher than at control temperature. Our data therefore indicate that rapid cooling has an important beneficial influence on the survival of *P. tricornutum* cells during a hypo-osmotic shock.

### Cold shock is associated with Ca^2+^-dependent K^+^ efflux

**Figure 9:**
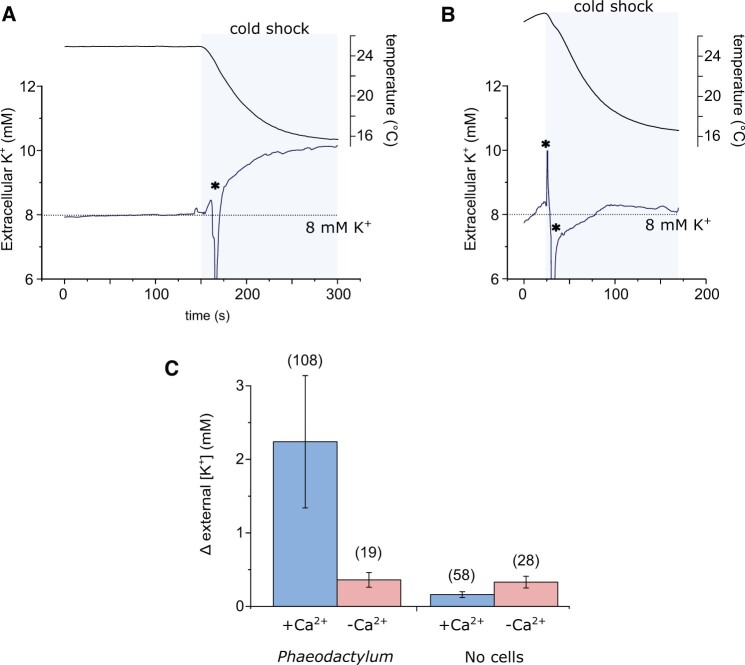
Cold shock induces a Ca^2+^-dependent K^+^ efflux. A, K^+^ efflux from *P. tricornutum* cells during a cold shock. A K^+^ microelectrode was placed adjacent to densely packed *P. tricornutum* cells to measure K+ in the immediate vicinity of the cell. A cold shock was applied by perfusion. The increase in extracellular K^+^ is the result of K^+^ efflux from the cells. The temperature in the dish is also shown (upper trace). B, Extracellular K^+^ during a cold shock in the absence of external Ca^2+^ (perfusion with ASW-Ca^2+^ + 200 μM EGTA). C, Mean change in extracellular K^+^ around *P. tricornutum* cells during a cold shock. “No cells” indicates control experiments where the experimental setup was identical, but no *P. tricornutum* cells were present in order to assess whether the performance of the K^+^ microelectrode was influenced by temperature. The total number of replicates for each treatment are shown in parentheses, error bars = se.

Ca^2+^-dependent K^+^ efflux plays an essential role in cellular volume control in *P. tricornutum* during hypo-osmotic shock (Helliwell et al., 2021b). We therefore tested whether the [Ca^2+^]_cyt_ elevations induced by cold shock also resulted in a K^+^ efflux that could influence cellular osmolarity. We settled a mono-layer of *P. tricornutum* cells onto a microscopy dish and used a K^+^-selective microelectrode to measure changes in extracellular K^+^ in the immediate vicinity of these cells. The cells were perfused with ASW at 25°C, before rapidly switching to 12°C. In each case, the cold shock induced a clear increase in extracellular K^+^ around the *P. tricornutum* cells (Figure 9, A–C). Application of a cold shock in the absence of external Ca^2+^ greatly reduced K^+^ efflux from the cells, indicating that the K^+^ efflux is Ca^2+^ dependent. Very little change in extracellular K^+^ was observed during a cold shock in the absence of cells, indicating that the performance of the K^+^-selective microelectrode was not affected by the change in temperature. We conclude that cold shock induces Ca^2+^-dependent K^+^ efflux in *P. tricornutum* cells, which may contribute to volume regulation during a simultaneous hypo-osmotic shock.

## Discussion

This study shows that transient [Ca^2+^]_cyt_ elevations are a consistent response to the rapid cold shocks likely to be experienced by intertidal diatoms. By using a continuous perfusion system, our study was able to avoid a shear-related [Ca^2+^]_cyt_ response, which may have masked a cold [Ca^2+^]_cyt_ response in earlier investigations using *P. tricornutum* expressing aequorin (Falciatore et al., 2000). The cold-induced [Ca^2+^]_cyt_ elevations are shown to be specifically involved in sensing the rate of cooling rather than the absolute temperature. A similar dependence of the amplitude of [Ca^2+^]_cyt_ elevations on the rate of cooling has been observed in Arabidopsis, which showed [Ca^2+^]_cyt_ elevations at cooling rates down to 0.05°C s^−1^ (Plieth et al., 1999), indicating greater sensitivity of Arabidopsis to slower cooling rates. *Phaeodactylum tricornutum* and *T. pseudonana* did not show a Ca^2+^ signaling response to rapid warming, suggesting that the Ca^2+^ signaling pathways of animals and plants in response to elevated temperatures are not conserved in diatoms. Diatoms therefore likely use alternative cellular mechanisms for thermosensation in response to rapid heat shock, although as only short-term temperature increases were evaluated in our study, we cannot rule out a potential role for Ca^2+^ signaling in response to longer-term temperature increases.

Our environmental data indicate that rapid cooling is likely to occur following tidal immersion on days when the air temperature is substantially warmer than the sea temperature. The rate of cooling will depend primarily on the volume of the water in each pool, the temperature difference between the pool and the incoming tide and the rate of immersion (e.g. wave action). Diatoms only exhibited [Ca^2+^]_cyt_ elevations in response to very rapid cooling (>1°C s^−1^) and so natural populations may not experience a sufficient rate of cooling if they are present in large volumes of water (e.g. deeper pools). However, diatoms are also abundant in many shallow environments in the intertidal zone, most notably in biofilms on the surface of tidal flats, rocks, or microalgae (Thompson et al., 2004). These low volume environments are likely to experience near instant changes in temperature following rapid tidal immersion.

Cold shock-induced [Ca^2+^]_cyt_ elevations in *P. tricornutum* do not play an obvious role in acclimation to low temperatures. We found no longer-term growth effects of experimentally blocking the cold shock Ca^2+^ signal. Cold signaling in *P. tricornutum* therefore differs from plants and insects (Knight and Knight, 2012; Teets et al., 2013), in which the [Ca^2+^]_cyt_ elevations play a direct role in acclimation to lower temperatures. The [Ca^2+^]_cyt_ response in *P. tricornutum* is specifically induced by rapid cooling, which points to a potential role in short-term regulation of cellular processes rather than longer term acclimation to a change in temperature. Of particular interest is the interaction between cold shock and osmotic shock, since intertidal organisms are often likely to experience these stresses simultaneously, during an incoming tide or rain precipitation (Lewin and Guillard, 1963; Kirst, 1990). Given the nature of the osmotic and cold shock Ca^2+^ signals identified in *P. tricornutum*, it is most likely that they involve distinct sensory pathways, as evidenced by their additive nature and the appearance of a biphasic [Ca^2+^]_cyt_ elevation when cells were treated with simultaneous cold and osmotic shocks. Whether these distinct responses represent Ca^2+^ entry through different Ca^2+^ channels or are due to sequential activation of the same Ca^2+^ channel by different stimuli with little or no refractory period remains to be determined, although it is worthy of note that both the osmotic (Helliwell et al., 2021b) and the cold-induced Ca^2+^ signals initiate at the cell apices (Figure 2B). Cold and osmotic Ca^2+^ signals also both require the presence of external Ca^2+^, indicating a shared requirement for plasma membrane Ca^2+^ channels, at least in the initiation of the [Ca^2+^]_cyt_ elevation.

The protective effect of cold shock on survival of *P. tricornutum* in response to severe osmotic shock may arise directly from cooperative Ca^2+^ signaling (Supplemental Figure S4). The hypo-osmotic shock-induced [Ca^2+^]_cyt_ elevations lead to rapid efflux of K^+^ in *P. tricornutum*, which restricts cell volume increase and prevents bursting (Helliwell et al., 2021b). The results here strongly suggest that cold-induced [Ca^2+^]_cyt_ elevations may also act directly to trigger K^+^ efflux from the cytosol, for example through the activation of Ca^2+^-dependent K^+^ channels. Whether the rapid loss of K^+^ plays a physiological role in acclimation to low temperature is unclear, but it would clearly serve to lower the osmolarity of the cell. Given the frequent co-occurrence of cold and hypo-osmotic shocks, the cold-induced [Ca^2+^]_cyt_ elevations may therefore function primarily to support osmoregulation. Rapid cooling does not appear to adversely harm the cell when Ca^2+^ signaling is inhibited, whereas a severe hypo-osmotic shock will lead to cell bursting within seconds if cell volume is not controlled (Helliwell et al., 2021b).

Osmoregulation in response to hypo-osmotic stress in diatoms (and most other eukaryotes) is most likely initiated by activation of mechanosensitive channels due to the increase in cell volume (Helliwell et al., 2021b). Mechanosensitive channels only activate when the membrane is under tension, that is, when swelling has already occurred, and cell viability is therefore under immediate threat if rapid osmoregulation cannot be achieved. The K^+^ efflux in response to a cold shock would allow the cell to reduce its osmolarity even if this critical increase in membrane tension is not perceived. By associating K^+^ efflux with an additional stimulus that commonly co-occurs with hypo-osmotic shock, diatoms can augment the osmoregulatory response and help prevent cell swelling to critical levels. Consistent with this hypothesis, cold-induced [Ca^2+^]_cyt_ elevations were only associated with very rapid cooling. A more gradual exposure to hypo-osmotic stress conveys a much lower risk of cell bursting, reducing the need to augment the osmoregulatory response.

We should also consider that low temperature may have a direct effect on reducing mortality during hypo-osmotic stress that is independent of the signaling component, for example by increasing cell wall rigidity. However, the protective effect of cold shock in the absence of Ca^2+^ was small compared to the much greater reduction in mortality in the presence of external Ca^2+^. We were unable to identify pharmacological inhibitors to selectively inhibit either osmotic or cold associated Ca^2+^ signaling and the removal of external Ca^2+^ completely inhibited both signaling pathways. Dissecting the individual contributions of these signaling pathways to cell survival during simultaneous shocks is therefore not currently easily achieved. Selective inactivation of the underlying molecular mechanisms through genetic approaches will most likely be required to fully understand the nature of the cross-talk between the signaling pathways.

Cellular responses to stressors are commonly examined in isolation in the laboratory in order to simplify the elucidation of the signaling pathways responsible. However, organisms often have to respond to inputs from multiple stimuli simultaneously in their natural environment, leading to cross-talk between signaling pathways. Cross-talk in cell signaling can occur when two distinct stimuli trigger a shared cellular response that confers tolerance to both stressors. This may involve activation of a common receptor or activation of independent receptors that converge on a specific node in the signaling pathway (Knight and Knight, 2001). Cross-talk with temperature sensing is likely to have evolved when another stress occurs simultaneously with temperature or with a predictable temporal link (i.e. one stimulus consistently precedes the other) (Sinclair et al., 2013). In the case of intertidal zone, many environmental parameters will exhibit a degree of covariance associated with tidal immersion and emersion. It seems likely that organisms inhabiting these environments have developed mechanisms of cross-talk in their pathways of environmental perception that enable them to optimize their physiological responses.

There are multiple examples of cross-talk between temperature and osmotic stress signaling pathways in other eukaryotes. In plants, freezing temperatures can lead to cellular water loss due to external ice formation and many of the genes within the cold-responsive (COR) regulon are also inducible by drought (Boyce et al., 2003). The cold-responsive C-repeat binding factors/dehydration-responsive element-binding (CBF/DREB1) and drought-responsive DREB2 transcription factors both bind to a common promoter element (DRE), leading to convergence of the cold and drought signaling pathways (Boyce et al., 2003). Overexpression of the cold-responsive DREB1A transcription factor in Arabidopsis resulted in enhanced tolerance to both freezing and drought stress (Liu et al., 1998). In addition, Arabidopsis plants treated with the phytohormone abscisic acid, which plays a primary role in drought tolerance, also show enhanced freezing tolerance (Mantyla et al., 1995). Cross-talk between temperature and osmotic stress signaling pathways have also been documented in yeast. *Saccharomyces cerevisiae* exhibits a high osmolarity (HOG) response to hyper-osmotic stress that results in increased production of the compatible solute, glycerol. The HOG response is mediated by a mitogen-activated protein kinase pathway that can also be activated by other stimuli including both cold and heat shocks. Heat shock activates the HOG pathway indirectly by stimulating loss of glycerol, leading to hyper-osmotic stress (Winkler et al., 2002; Dunayevich et al., 2018).

Our results indicate that cross-talk between Ca^2+^-mediated cellular signaling mechanisms is an important consideration in the response of marine organisms to multiple stressors. While our results are discussed primarily in the context of the intertidal zone where rapid substantial changes in temperature are a regular occurrence, the conserved nature of cold-induced Ca^2+^ signaling in *T. pseudonana* suggests that this pathway may be important more widely in diatom ecology. The cold-induced [Ca^2+^]_cyt_ elevations in *T. pseudonana* exhibit different characteristics from *P. tricornutum* that may reflect differences in their physiological response. Planktonic diatoms will undoubtedly encounter substantial fluctuations in temperature and salinity in near-shore and estuarine environments or when they are mixed through the thermocline, although the magnitude and rate of the temperature changes are likely to be lower. Diatoms inhabiting sea ice environments may also experience rapid changes in temperature and salinity, for example, during flushing of hyper-saline brine channels with melt water (Mock and Junge, 2007). Future elucidation of the mechanisms of cross talk in these signaling pathways will allow us to understand how diatoms successfully integrate inputs from multiple environmental stimuli, which has likely played a major role in their success in diverse and highly dynamic environmental regimes.

## Materials and methods

### Recording of rockpool temperature

Temperature data were recorded using a 27-mm Envlogger v2.4 (ElectricBlue, Porto, Portugal) encased in acrylic resin, recording in 30-min intervals with a resolution of 0.1°C. The Envlogger was secured to the substrate using Z-Spar A-788 epoxy resin roughly 3 cm below the surface waters of a shallow mid-shore rockpool measuring ∼8-cm deep at Looe Hannfore, Cornwall, UK (50.3411, −4.4598) from July 1, 2019 to July 7, 2019.

### Strains and culturing conditions

The wild-type *P. tricornutum* strain used in this study was CCAP 1055/1 (Culture Collection of Algae and Protozoa, SAMS, Scottish Marine Institute, Oban, UK). A *P. tricornutum* strain transformed with the R-GECO1 Ca^2+^ biosensor (PtR1) was generated as described previously (Helliwell et al., 2019). Three *eukcata1* knockout strains in the PtR1 line (labeled A3, B3, and B6) were generated by CRISPR–Cas9-mediated gene editing, with two single-guide RNAs ∼50-bp apart targeted to the pore region of *PtEUKCATA1* resulting in deletions of 38–124 bp (Helliwell et al., 2019). The *T. pseudonana* strain expressing the R-GECO1 biosensor (TpR1) was generated as described in Helliwell et al (2021a, 2021b). Cultures were maintained in natural seawater with f/2 nutrients (Lewin and Guillard, 1963; Guillard, 1975); modified by the addition of 106-μM Na_2_SiO_3_.5H_2_O and the exclusion of vitamins (*P. tricornutum* only). For imaging experiments, cells were acclimated to an ASW medium for minimum 10 days prior to analysis. ASW contained 450-mM NaCl, 30-mM MgCl_2_, 16-mM MgSO_4_, 8-mM KCl, 10-mM CaCl_2_, 2-mM NaHCO_3_, 97-µM H_3_BO_3_, f/2 supplements, and 20-mM HEPES (pH 8.0). Cultures were grown at 18°C with a 16:8 light/dark cycle under illumination of 50 µmol m^−2^ s^−1^.

### Epifluorescence imaging of R-GECO1 fluorescence

Cell culture measuring 500 µL of was added to a 35-mm microscope dish with glass coverslip base (In Vitro Scientific, Sunnyvale, CA, USA) coated with 0.01% poly-L-lysine (Merck Life Science UK, Gillingham, Dorset) to promote cell adhesion to the glass surface. Cells were allowed to settle for 5–20 min at RT under light. R-GECO1 was imaged using a Leica DMi8 inverted microscope (Leica Microsystems, Milton Keynes, UK) with a 63 × 1.4NA oil immersion objective, using a Lumencor SpectraX LED light source (4% intensity) with a 541–551-nm excitation filter and 565–605-nm emission filter. Images were captured with a Photometrics Prime 95B sCMOS camera (Teledyne Photometrics, Birmingham, UK) with a 300-ms exposure. Images were captured at 3.33 frames per second using Leica application suite X-software v.3.3.0.

### Administration of temperature shocks to cells in the imaging setup

The dish was perfused with ASW without f/2 nutrients at a standard flow rate of 16 mL min^−1^. To achieve rapid changes in temperature in the dish, the perfusion was switched between solutions of different temperature to achieve target temperatures of approximately 10°C, 22°C, or 30°C respectively. Actual dish temperature was recorded using a Firesting micro optical temperature sensor (Pyroscience GmbH, Aachen, Germany). For the majority of experiments, cells were perfused with warmer media (dish temperature 30°C) for 1 min prior to application of the cooling shock (these conditions reflect those observed in the rockpool observations). The perfusion flow rate was altered to achieve different temperature change rates. As cooling rate was not linear, the maximum cooling rate was defined as the largest decrease temperature within a one second period.

### Application of inhibitors and elicitors

External Ca^2+^ was removed by perfusion with ASW without CaCl_2_ containing 200-μM EGTA. RR was added to cells at a final concentration of 10 µM, 5 min prior to cold shock treatment. Menthol was prepared as a 1-M stock solution in DMSO and used at concentration of 1 mM, resulting in a final DMSO concentration of 0.1% v/v.

### Processing of imaging data

Images were processed using LasX software (Leica). The mean fluorescence intensity within a region of interest (ROI) over time was measured for each cell by drawing an ROI encompassing the whole cell. Background fluorescence was subtracted from all cellular *F*-values. The change in the fluorescence intensity of R-GECO1 was then calculated by normalizing each frame by the initial value (*F/F*_0_). [Ca^2+^]_cyt_ elevations were defined as any increase in *F/F*_0_ above a threshold value (1.5). The duration of a [Ca^2+^]_cyt_ elevation was defined as the peak width at half maximal amplitude. To visualize the spatial distribution of a [Ca^2+^]_cyt_ elevation, each frame was divided by a corresponding background image generated by applying a rolling median (30 frames) to the image-series (Image J). The resultant time series images were pseudo-colored to indicate changes in fluorescence.

### Statistical analysis

Graphs and statistical analyses were performed using Sigmaplot v14.0 (Systat Software, Slough, UK). Error bars represent standard error of the mean. Unless indicated otherwise, imaging experiments were repeated three times with independent cultures on different days to ensure reproducibility of the response.

Normal distribution of respective datasets was tested using Shapiro–Wilk’s normality test. When passed, statistical analysis of datasets with two groups were done with Student’s *t* test, and when not passed with Mann–Whitney’s rank sum test. Statistical analysis of datasets with more than two groups were performed using an analysis of variance (ANOVA) followed by a Holm–Sidak post hoc test when the normality test was positive. When the normality test was negative, Kruskal–Wallis’ one-way ANOVA on Ranks was used instead. All statistical tests were performed with Sigmaplot v14.0.3.192 (Systat software Inc).

### Growth at different temperatures after a cold shock

For the growth curves, cells were grown to mid exponential phase (2.73 × 10^6^ cells mL^−1^). The culture was divided into 10-mL aliquots and cells were pelleted by centrifugation (1250 *g* at 18°C). Cells were washed in 40-mL ASW ± Ca^2+^ and pelleted again by centrifugation. Cells were then resuspended in their respective treatments (20 mL of ASW ± Ca^2+^ at 18°C or 4°C to administer a rapid cold shock). The temperature of the ASW was monitored after mixing and found to have remained at 4°C. After 10 min, 2 mL of each culture was used to inoculate culture vessels containing 18 mL of standard ASW F/2 media containing 10-mM Ca^2+^ (approximately starting density of 6.8 × 10^4^ cells mL^−1^) and cultures were grown at 18°C or 4°C for 5 days or 30 days, respectively. The cells were therefore only in media without Ca^2+^ for 10 min, which acts to inhibit Ca^2+^ signaling during rapid cooling but avoids prolonged exposure to very low external Ca^2+^.

### Cell survival during hypo-osmotic shock

To examine the effect of temperature on cell viability during hypo-osmotic shock, 10 mL of a late log phase culture (6 × 10^6^ cells mL^−1^) were pelleted by centrifugation (1250 *g* at 18°C). Cells were washed twice with 10-mL ASW ± Ca^2+^ and 250-μL aliquots were taken. To apply the hypo-osmotic and cold shock treatments, 750 µL of ASW (±Ca^2+^) or deionized water at two different temperatures (20°C or 4°C) were added to each tube. Addition of water results in a severe hypo-osmotic osmotic shock (final concentration 25% ASW) simultaneously with the temperature shock. The cells were then incubated at their respective temperatures for 3 min prior to addition of 5-μM Sytox Green (Thermo Fisher Scientific, Loughborough, UK). All treatments were then incubated at 20°C 15 min in darkness. Cell viability was measured with a LUNA-FL Dual Fluorescence Cell Counter (Logos Biosystems, Villeneuve d’Ascq, France) to count live (displaying red chlorophyll fluorescence) versus dead cells (Sytox Green stain) with following settings: Excitation intensity green = 11, red = 7, count threshold for both = 3.

### Quantification of K^+^ efflux in *P. tricornutum* populations using K^+^-selective microelectrodes

K^+^ microelectrodes were fabricated as described previously (Helliwell et al., 2021b). Clark GC-1.5 borosilicate glass capillaries (Harvard Apparatus, Cambridge, UK) were pulled to a fine point using a P-97 pipette puller (Sutter, Novato, CA, USA). The pipette tips were then gently broken to produce a diameter of ca 10–20 µm. The capillaries were salinized by exposure to N,N-dimethyltrimethylsilylamine (TMSDMA) vapor at 200°C for 20 min within a closed glass Petri dish. The K^+^ microelectrodes were prepared by introducing K^+^ ionophore I (Sigma Aldrich, Gillingham, Dorset, UK) into the pipette tip by suction. Pipettes were then back-filled with the filling solution (100-mM NaCl, 20-mM HEPES pH 7.2, and 10-mM NaOH). The reference electrode was filled with 3-M KCl, and data were recorded using an AxoClamp 2B amplifier, with pClamp v10.6 software (Molecular Devices, CA, USA). Each K^+^ microelectrode was calibrated using a two-point calibration with standard KCl solutions. The mean slope of the calibrated electrodes was 53.0 ± 1.3 mV per decade (±se).

For the measurements, 10 mL of *P. tricornutum* cells from exponential culture containing 10^6^–10^7^ cells mL^−1^ were centrifuged at 1250 *g* for 10 min and re-suspended in 1 mL of ASW. The cells were then allowed to settle on a poly-L-lysine coated microscope dish. Cells were perfused with ASW or ASW-Ca^2+^ (0-μM Ca^2+^ + 100-µM EGTA) and cold shocks were applied as described for the microscopy observations. Control experiments were performed in the absence of *P. tricornutum* cells to ensure that the change in temperature did not alter the performance of the K^+^ microelectrodes.

## Supplemental data

The following materials are available in the online version of this article.

**Supplemental Figure S1.** [Ca^2+^]_cyt_ elevations in response to repeated cold shocks.

**Supplemental Figure S2.** Cold shocks from different starting temperatures.

**Supplemental Figure S3.** Cell volume during cold shock.

**Supplemental Figure S4.** Proposed Ca^2+^ signaling pathways in response to osmotic and cold stress.

## Funding

The work was supported by a European Research Council Advanced Grant to CB (ERC‐ADG‐670390) and a Natural Environment Research Council award to GLW (NE/V013343/1).

*Conflict of interest statement*. None declared.

## Supplementary Material

kiac324_Supplementary_Data

## Notes

C.B., F.H.K., and G.L.W. conceived the study. F.H.K. performed the majority of the experimental analyses, including all imaging experiments. K.E.H. contributed to measurements of osmotic stress. A.C. performed K^+^ microelectrode measurements. H.P.W. and N.M. performed the environmental monitoring. T.M., A.H., and T.G. contributed to the transformation of *T. pseudonana* with R-GECO1. G.L.W., C.B. and F.H.K. analyzed the data and wrote the manuscript.

The author responsible for distribution of materials integral to the findings presented in this article in accordance with the policy described in the Instructions for Authors (https://academic.oup.com/plphys/pages/general-instructions) is Glen Wheeler (glw@mba.ac.uk).
