# Supplementary material for: Cold-induced [Ca2+]cyt elevations function to support osmoregulation in marine diatoms
Source: Plant Physiol. 2022 Jul 27;190(2):1384–99. doi: 10.1093/plphys/kiac324 (PMC9516774; doi:10.1093/plphys/kiac324)
Supplement: kiac324_Supplementary_Data [file kiac324_supplementary_data.pdf]

Supplemental Information

Supplemental Figure S1: $[Ca^{2+}]_{cyt}$ elevations in response to repeated cold shocks

Supplemental Figure S2: Cold shocks from different starting temperatures.

Supplemental Figure S3: Cell volume during cold shock

Supplemental Figure S4: Proposed Ca^{2+} signalling pathways in response to osmotic and cold stress.

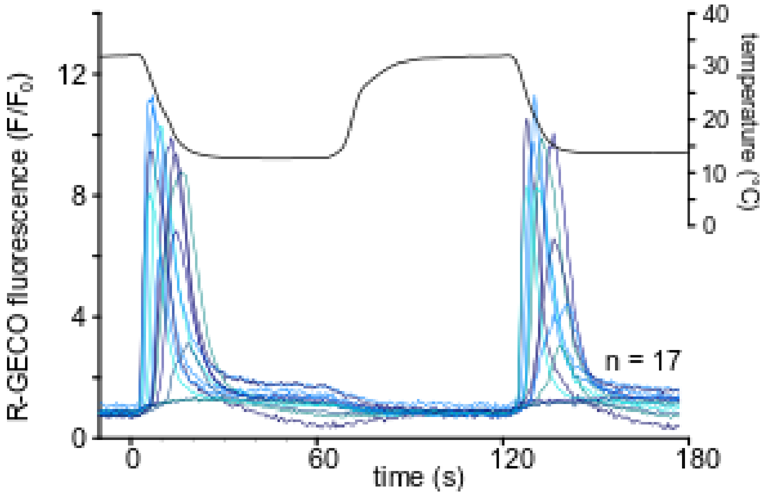

Supplemental Figure S1: $[Ca^{2+}]_{cyt}$ elevations in response to repeated cold shocks. Representative fluorescence ratio traces from PtR1 cells in response to two sequential cold shocks (30 °C to 12 °C). Cells show a very similar response to the second cold shock at 120 s, although there is no $[Ca^{2+}]_{cyt}$ elevation in response to rapid warming at 60 s.

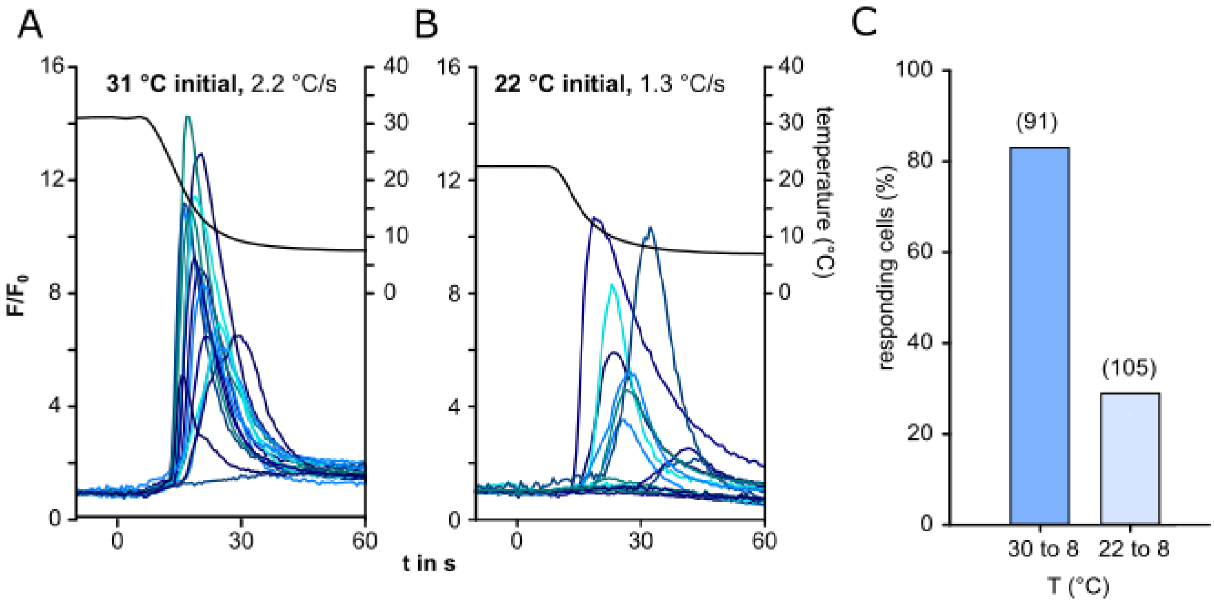

Supplemental Figure S2: Cold shocks from different starting temperatures. A) Cold-shock induced $[Ca^{2+}]_{cyt}$ elevations displayed by R-GECO1 cells following perfusion of cold ASW to cells at an initial temperature of 30 °C. 25 representative traces are shown. **B)** Cold-shock induced $[Ca^{2+}]_{cyt}$ elevations displayed by R-GECO1 cells following perfusion of cold ASW to cells at an initial temperature of 22 °C. Note that the maximum rate of cooling was lower than (A) due to the lower initial temperature. 25 representative traces are shown. **C)** Percentage of cells demonstrating a $[Ca^{2+}]_{cyt}$ elevation in the experiments shown in (A) and (B). Number of cells examined is shown parentheses.

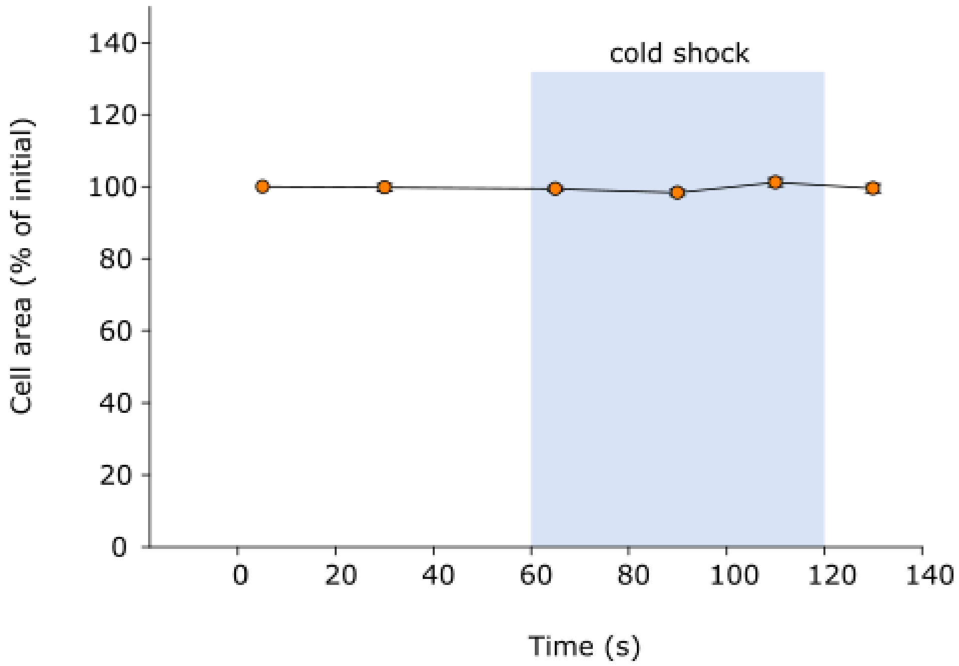

Supplemental Figure S3: Cell volume during cold shock. The mean area of PtR1 cells measured during cold shock (30 to 10 °C, max. 3 °C s^{-1}) using light microscopy. No increase in cell area was observed, indicating that cell volume did not increase during the cold shock. n = 13 cells. Error bars represent SE (bars are smaller than symbols).

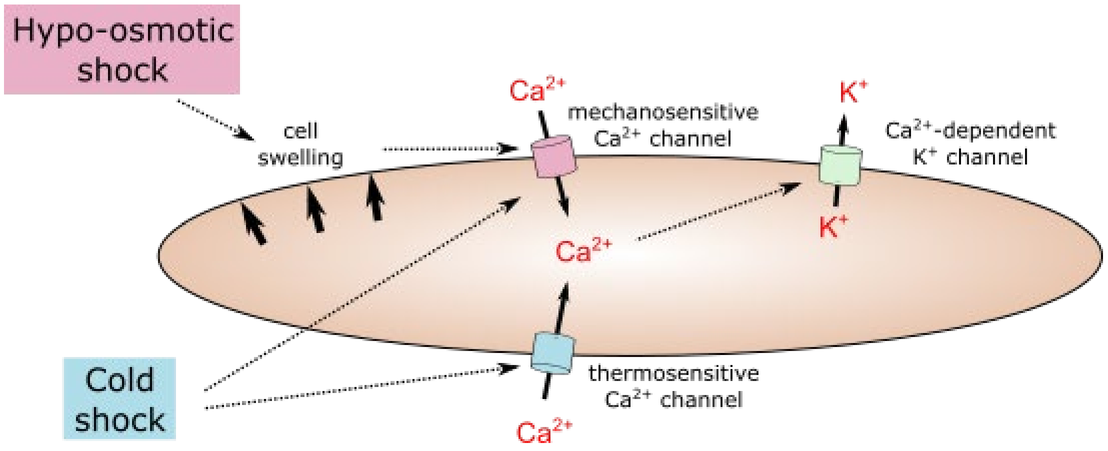

Supplemental Figure S4: Proposed Ca^{2+} signalling pathways in response to osmotic and cold stress. Schematic representation of cross-talk between hypo-osmotic and cold-shock signalling pathways. Application of a hypo-osmotic shock (e.g. seawater of lower salinity) results in cell swelling, activating mechanosensitive Ca^{2+}-permeable channels in the plasma membrane. The resultant increase in cytosolic Ca^{2+} $[Ca^{2+}]_{cyt}$, leads to K^+ efflux from the cell, either through the activation of Ca^{2+}- dependent K^+ channels (as shown in the schematic) or via an indirect mechanism. Rapid cooling causes an increase in $[Ca^{2+}]_{cyt}$ by activation of a Ca^{2+}-permeable channel in the plasma membrane. This may either be due to temperature sensitivity of the same mechanosensitive ion channel required for the osmotic shock response or through activation of a separate thermosensitive Ca^{2+}-permeable channel. As with hypo-osmotic shock, the rise in $[Ca^{2+}]_{cyt}$ leads to K^+ efflux.
